# Insights on the NF-κB System Using Live Cell Imaging: Recent Developments and Future Perspectives

**DOI:** 10.3389/fimmu.2022.886127

**Published:** 2022-06-29

**Authors:** Cise Kizilirmak, Marco E. Bianchi, Samuel Zambrano

**Affiliations:** ^1^ School of Medicine, Vita-Salute San Raffaele University, Milan, Italy; ^2^ Division of Genetics and Cell Biology, IRCCS San Raffaele Scientific Institute, Milan, Italy

**Keywords:** NF-kappa B (NF-κB), live cell imaging, dynamics, biological oscillators, inflammation, immune response

## Abstract

The transcription factor family of nuclear factor kappa B (NF-κB) proteins is widely recognized as a key player in inflammation and the immune responses, where it plays a fundamental role in translating external inflammatory cues into precise transcriptional programs, including the timely expression of a wide variety of cytokines/chemokines. Live cell imaging in single cells showed approximately 15 years ago that the canonical activation of NF-κB upon stimulus is very dynamic, including oscillations of its nuclear localization with a period close to 1.5 hours. This observation has triggered a fruitful interdisciplinary research line that has provided novel insights on the NF-κB system: how its heterogeneous response differs between cell types but also within homogeneous populations; how NF-κB dynamics translate external cues into intracellular signals and how NF-κB dynamics affects gene expression. Here we review the main features of this live cell imaging approach to the study of NF-κB, highlighting the key findings, the existing gaps of knowledge and hinting towards some of the potential future steps of this thriving research field.

## Introduction

It is hard to envision a more dynamic process than the immune response to pathogens or damage in multicellular organisms (1), a complex choreography underpinned by a remarkably conserved transcriptionally mediated regulation (2). Key steps of this process are coordinated by the family of transcription factors (TFs) of the nuclear factor kappa B (NF-κB) proteins: a family of dimeric TFs that control a wide variety of transcriptional processes in innate and adaptive immune responses (3, 4). A key aim of this review is to highlight how live cell imaging has put in center stage the dynamic nature of NF-κB in controlling inflammation and the immune response.

**Figure 1:**
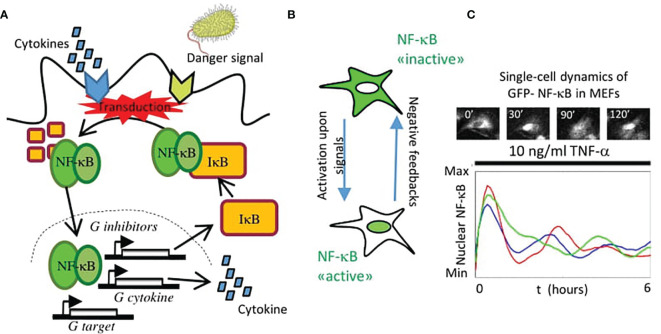
The nuclear localization dynamics of NF-κB. **(A)** Scheme of the activation of NF-κB and its regulation in the canonical pathway: an external signal (cytokine or pathogen-associated) is transduced and leads to the degradation of the inhibitors IκB that keep NF-κB sequestered in the cytosol. Then it translocates into the nucleus, where it activates the expression of feedbacks that modulate the nuclear localization (e.g., genes that encode for the inhibitors IκB), a variety of cytokines and chemokines and other target genes. **(B)** In this way, activation of NF-κB is largely mediated by its localization into the nucleus that is counteracted by different negative feedback mechanisms. **(C)** When monitored in single cells, the nuclear localization of NF-κB can be oscillatory upon TNF-α. In the subplot, a representative image of a Mouse Embryonic Fibroblast (MEF) expressing knocked-in GFP fused to NF-κB upon TNF-α. Each line in the plot represents a different cell in the same population under the same treatment.

NF-κB was discovered as a DNA binding protein in activated B cells, where it plays a role in their maturation (5). Later NF-κB was found to be expressed in almost all cell types and shown to be a central regulator of the immune and inflammatory responses (6); one of its discoverers suggested half-jokingly that if he had imagined this, he would have chosen a simpler nomenclature for it (6). The NF-κB family is complex, not only in name: it is actually a family of dimeric TFs resulting from the combination of 5 monomers, p65(RelA), c-Rel and RelB, that contain a transactivating domain (TAD) and p52, p50 monomers, that do not. Dimers containing the p65 monomer are those with the strongest transcription activation potential (7) and are embedded in the so-called “canonical pathway” (**Figure 1A**); given the prevalence of p65-including dimers in the studies discussed in this work, we will refer to them as NF-κB unless otherwise stated (further details on the studies characterizing other subunits will be provided in the last section of this review). NF-κB is activated through a number of crucial receptors of pathogen associated molecular patterns (PAMPs) or damage-associated molecular patterns (DAMPs) by cells of the innate immune system, as a result of which it controls the expression of key inflammatory cytokines (8). Of note, NF-κB activation has been connected to more complex immunological mechanisms such as dendritic cell maturation (9), neutrophil recruitment (10), M1 polarization of the macrophages (11) and differentiation and activation of inflammatory T cells (12), a role that keeps emerging even in recent unbiased screenings (13). Furthermore, besides the central role in the response to pathogens, NF-κB plays a fundamental role in the processes of proliferation (14), apoptosis (15, 16), growth, differentiation, and morphogenesis of a variety of tissues (17–20). For all these reasons, when deregulated, NF-κB has been found to be linked with inflammatory diseases (6) and to act as the matchmaker in the complex interplay between cancer and inflammation (21).

Hence, it is unsurprising that this system requires a tight regulation, whose fundamental ingredients were dissected in the seminal paper by David Baltimore and his associates (22) and is schematically depicted in **Figure 1A**. In the inactive state, NF-κB is kept in the cytosol by its inhibitors IκB. Upon activation through external signals, such as tumor necrosis factor alpha (TNF-α), a signaling cascade leads to kinase-mediated inhibitor’s degradation allowing NF-κB localization in the nucleus (**Figure 1B**), where it regulates the expression of its target genes. These include, notably, its own IκB inhibitors, which bring NF-κB back to the cytosol and result in an effective negative feedback system (**Figures 1A, B**). Importantly, IκB inhibitors are fundamental but they are not the only transcriptional targets of NF-κB that regulate this circuit. These also include the zinc finger protein A20 which has both ubiquitin ligase and deubiquitinase activities that terminate the activation of the kinases leading to IκB degradation (23).

The global NF-κB negative feedback loop of course is dynamic, but only with the advent of fluorescent proteins it was possible to image the NF-κB dynamic behavior that it produces in single cells. In their pioneering work, Nelson and collaborators fluorescently tagged NF-κB and found that its nuclear localization dynamics upon TNF-α was very rich and included consistent oscillations in the nuclear concentration with a period close to 1.5 hours (24); an example is shown in **Figure 1C** for mouse embryonic fibroblasts (MEFs). Such oscillations were in principle possible, as suggested by detailed mathematical models of the NF-κB signaling network (22) but they were controversial at the beginning (25, 26). However, this pioneering study shed light on a fundamental truth: that NF-κB dynamics activation is complex and heterogeneous across cells and cannot be simply classified as an “on-off” process. Indeed, NF-κB has become a distinguished member of an ever-growing list of “pulsing” (if preferred to “oscillating”) genetic circuits studied through live-cell imaging (27), a list that includes genes in the circadian clocks (28), transcriptional regulators such as p53 (29), extracellular signal regulated kinases (Erk) (30), nuclear factor of activated T-cells (NFAT) (31), the mechanotransducer YAP/TAZ (in the Hippo pathway) (32) and the developmental clock Hes7 (33), to cite a few. The emerging view from these studies is that the rich TF dynamics observed through live cell imaging is not merely a by-product of their regulatory mechanisms (that typically also include negative feedbacks), but has a functional role in proper gene expression (34). Probably the research carried out on the NF-κB system provides the clearest examples of the importance of a “dynamic point of view” to understand the role of TFs, as we discuss in the present review.

**Figure 2:**
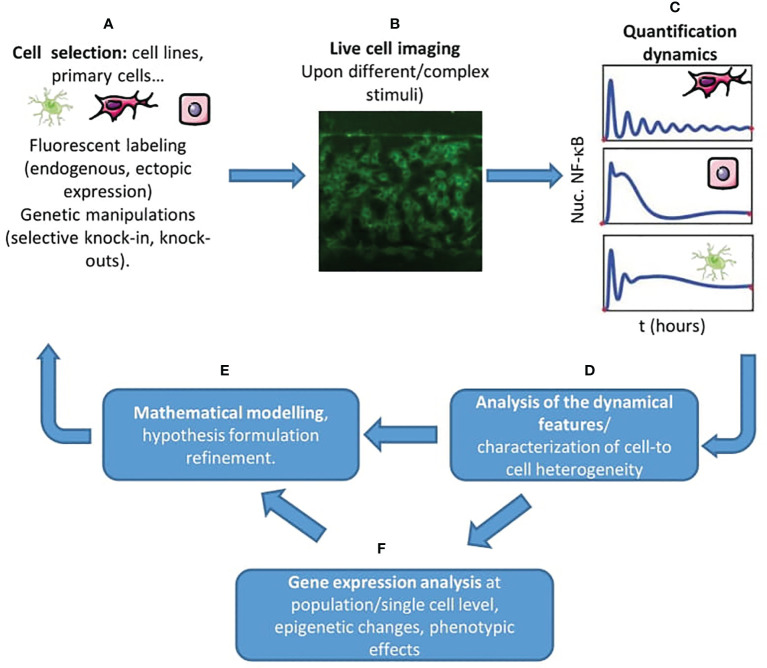
A live cell imaging centered workflow to unravel new features of NF-κB regulation. **(A)** The starting point is the proper selection of the cell system(s) of interest, the appropriate tagging, and the activating stimulus of interest, combined with the necessary genetic modifications allowing us to test our hypotheses. **(B)** Through live cell imaging of hundreds of cells- also in complex and carefully controlled microenvironments, e.g. using microfluidics (in the picture, MEFs plated in a gradient microfluidic chamber) - we can probe the dynamic response of NF-κB. **(C)** We can extract the dynamics of NF-κB upon our stimulus of interest for different cells and cell types. **(D)** Then, it is possible to extract the dynamical features that characterize the cell’s response in the experiment (maximum response, timing, oscillatory peaks) and characterize the heterogeneity of the population. **(E)** The use of mathematical models is crucial to characterize to what extent the experiments match our hypothesis, to formulate new ones and to inform new experiments. **(F)** We can also use gene expression analysis, evaluate phenotypic changes and epigenetic changes to complement our view of the regulation of NF-κB in the system considered.

Here, we aim to provide an overview on how live cell imaging has contributed to deepen our understanding of NF-κB role and regulation. To do so, we start by describing the methodologies used in the context of this approach (**Figure 2**). Next, we provide an overview of the findings that this approach has provided: on the heterogeneity of NF-κB dynamics within and between distinct cell populations; on how its activation is controlled by upstream and downstream regulators and on how NF-κB controls gene expression. Finally, we speculate about potential new directions in this research field.

## Key Methodologies in Live Cell Imaging-Based Studies of NF-κB

We will first discuss the main tools used to study the role of NF-κB dynamics in single living cells. The selection of the adequate cellular system and fluorescent tagging scheme is fundamental (**Figure 2A**). NF-κB was visualized for the first time in single cells by transient transfection with GFP-tagged p65, which allowed to dissect also the interaction with its inhibitors (35–37). These studies were performed in easy-to-handle cell lines, as in the pioneering work of Ref. (24) where oscillations of fluorescently tagged ectopically expressed NF-κB were observed in HeLa (human cervical carcinoma) and SK-N-AS (human S type neuroblastoma) cells. Although the use of ectopic expression is widely accepted today (38–40) it was received with some reticence by the community, given the possibility of artifacts due to overexpression. Several clever approaches have been proposed to circumvent these difficulties. For example, a possibility is to select clonal populations of lentiviral-infected cells where it is possible to know the ratio between the endogenous and the fluorescently tagged levels of expression of p65 (39). Another approach followed for instance with 3T3 cells was to knock-out p65 and transduce them to express fluorescently tagged p65 (41, 42). Cells from transgenic mouse models where the transgene expresses a fluorescently tagged p65 (43) should provide a nearly constant ratio between the tagged and the untagged endogenous p65. Finally, knock-in mouse models where a fluorescent protein is fused to the N-term of p65 allows the observation of NF-κB dynamics in primary cells, including mouse embryonic fibroblasts (44, 45) and bone-marrow derived macrophages (44, 46), circumventing the potential distortion of NF-κB signaling that might be present in cancer cell lines (47). A systematic comparison between all these tagging schemes, though, is missing.

Imaging (**Figure 2B**) is usually performed with confocal or widefield microscopes equipped with temperature and CO_2_ control, allowing imaging for more than 10 hours. A fundamental tool is the use of image analysis software able to extract NF-κB dynamics of hundreds of cells in an automatic way (**Figure 2C**); there is no gold standard in this context but different groups have made available their routines on public repositories or (more often) upon request. In most of the cases, these routines perform a segmentation of the cells’ nuclei that relies on the use of a fluorescent DNA dye like Hoechst (48) or of a fluorescent nuclear protein like H2B-GFP (41). Segmentation of the nucleus is typically easily achieved using a threshold on the nuclear signal intensity combined with watershed transformations. In some cases, the cytoplasm of each cell can also be segmented using a similar principle; indeed, to characterize the degree of nuclear localization different approaches are used such as quantifying the total nuclear intensity, the nuclear to total (nuclear plus cytosolic) intensity or the nuclear to cytosolic intensities; the latter internally normalized quantifications are recommended in situations where a stable proportionality between intensity and protein amount is not guaranteed. For the tracking, which is performed by connecting images of cells in consecutive frames, typically a nearest-neighbor approach works nicely although there are other more efficient algorithms, such as the Hungarian linker (49). From dynamic profiles of nuclear localization we can extract different quantifiers, starting from simple ones such as the maximum response, the timing, the area under the curve and the fold-change in nuclear level upon stimulus (**Figure 2D**) see e.g. (42, 45).

Finally, a key methodology complementary to live cell imaging is the extensive use of mathematical models (**Figure 2E**). For interested readers, introductions to the mathematical modeling of NF-κB are available (50, 51). In short, these mathematical models reproduce the time evolution in different biochemical compartments of the copy numbers/concentrations of biochemical species including NF-κB, its complex with the inhibitors, the target genes mRNA levels, etc… that react to give different products according to certain rates. Hence, the global temporal evolution of the system of biochemical molecules that form the NF-κB system is described typically by a set of ordinary differential equations although other approaches are also possible (52) that are typically solved numerically using appropriate softwares, and provide simulations of the dynamics of nuclear localization upon stimuli. In such models, the aim is to use a number of variables that is a reasonable compromise between detail in the description and mathematical simplicity. The latter is desired for many reasons, starting with the fact that many of the biochemical rates of the key processes of the NF-κB regulatory circuit are not known exactly. As an example of this search of balance, in the pioneering work (22) the synthesis of the three inhibitors IκBα, IκBβ;, IκBε, is modeled in detail since it serves the purposes of the paper: showing that partial knockdown of each of the inhibitor genes leads to different dynamics. However, in other works [e.g. (48, 53)] only the feedback of IκBα, alone or in combination with A20, is explicitly modeled, and this was enough to reproduce the phenomena observed.

Importantly, most of the mathematical models used in the works reviewed here reproduce, with different degrees of complexity, the same core mechanism roughly depicted in **Figure 1A**: NF-κB import to the nucleus is blocked by the binding of the IκB inhibitors, which mask its nuclear localization signal (3); following kinase-driven degradation of the IκBs, NF-κB nuclear localization is possible and its transport is enhanced. However, the synthesis of newly transcribed inhibitors upon NF-κB nuclear localization creates a pool of IκBs that gradually form new complexes with NF-κB leading to its re-localization in the cytoplasm, where this cycle might start again (depending of other factors, such as the persistence of the external stimulus and on the action of other negative feedbacks, such as A20). As we will discuss below, this might be an oversimplification of the actual mechanisms in place but this kind of mathematical models can provide informed predictions that can be subsequently tested experimentally and lead to novel insights on NF-κB regulation.

## Insights on NF-κB Response to Stimuli: Heterogeneity With Precise Rules

With these methodologies in mind, we can provide an overview of the key observations and insights obtained from live cell imaging of the NF-κB system. A first straightforward observation is that, upon activation, NF-κB follows a nuclear localization dynamics that is heterogeneous within the cell population but also different between cell types. This is somehow puzzling considering the key roles of NF-κB in dictating the cell’s transcriptional program in different fundamental processes in health and disease, and all the more so when it has been proposed that this very dynamic mode of action allows an improved information transmission in comparison to non-dynamic responses (54). In this section we describe how can NF-κB dynamics be heterogeneous and yet obey precise rules.

First, NF-κB dynamics was shown to be qualitatively different between cell lines. The work of Nelson et al. (24) already showed how continuous exposure to TNF-α results in oscillations with a time period of 100 minutes in SK-N-AS cells, but less pronounced oscillatory behavior in HeLa cells, as confirmed by others (39, 55). Indeed, a study carried out by transducing different cancer cell lines showed how their dynamics upon TNF-α could differ (56). This is not only the case for cancer cells: 3T3 cells display sustained NF-κB oscillations upon TNF-α (53), while in immortalized MEFs we observed damped oscillations with few oscillatory peaks and a fraction of non-oscillatory cells (48); mathematical models indeed show that different behaviors are possible depending on the emergence of a Hopf bifurcation (a critical point where a system’s stability switches) (48). The activating stimulus also plays a role: LPS has been shown to induce a persistent and non oscillatory nuclear localization of NF-κB in RAW cells (57) and primary bone marrow derived macrophages (46) although oscillations were reported by others (43, 58); a more persistent nuclear localization was also reported for 3T3 cells upon LPS as a result of autocrine-paracrine TNF-α signaling (41), which induces a secondary activation that leads to a stronger and more long-lasting NF-κB activation. This difference in the response hints towards stimulus specificity; in fact, it has been shown recently that macrophages and 3T3 cells discriminate between different pathogen-derived stimulations and cytokines through different NF-κB dynamical features (59, 60).

Beyond the cell and stimulus specificity of NF-κB dynamics, even within homogeneous cell populations the dynamics can be quite heterogeneous. However, this heterogeneity has been shown to follow precise rules. For example, independent studies show that upon increasing doses of TNF-α an increasingly high fraction of cells respond (42, 61); mathematical models including stochasticity in the activation of the receptors and negative feedbacks were able to reproduce this heterogeneous behavior (42). Using a microfluidics device, it was shown that a long and weak stimulation leads to NF-κB activation of fewer cells in comparison to a short but strong stimulus pulse (62); these works confirm the existence of an activation threshold that relies on cellular noise. Subsequent studies using microfluidics have instead proposed that the activation threshold varies between single cells in the population (63). Other dynamical features emerge as robust in spite of the heterogeneity of the population. In a study that combined live cell imaging with mathematical modeling it was reported that the oscillations period (measured as inter-peak timing) remains constant in the population upon different stimuli, even if it can vary within the same cell (64). Finally, other sources of cell-to-cell variability have been precisely identified, for example E2F activity during the cell cycle (65).

Overall, it is becoming clear that NF-κB responds heterogeneously to stimuli within a cell population, but such heterogeneity follows precise rules that can lead to insights on how cell populations respond collectively to inflammatory cues.

## Insights on the Regulation of NF-κB Activity: How the Interplay Between Regulatory Elements Defines Dynamic Signals

An important advantage of single-cell live measurements of NF-κB is that they also allow us to precisely monitor how NF-κB responds to different stimuli. This has contributed to improve our understanding on how the activation of NF-κB is regulated by the interaction of ligands with receptors, by upstream transducers and by downstream negative feedbacks (**Figure 1A**).

The paper by Ashall et al. (38) is a clear example of how imaging allowed to dissect the role of negative regulators of NF-κB activity: it was shown that pulses of TNF-α followed by washouts led to nuclear localizations of NF-κB of decreasing amplitude whose frequency roughly matched that of the periodic stimulation. The decrease in the amplitude disappeared if the timing between pulses was long enough, suggesting a “refractory time” of the system. Mathematical models and experiments showed the key role of the interplay between A20 and the two negative feedbacks IκBα and IκBε in this refractoriness; in particular, they show that the persistence of significant levels of the kinase-inhibiting A20 protein led to weaker responses to subsequent TNF-α pulses. Of note, a follow-up study (66) indeed showed that the refractoriness was lost if il-1β was used as a subsequent stimulus instead of TNF-α, demonstrating the stimulus-specificity of this dynamical feature. The importance of the interplay between feedbacks was also been highlighted by a number of studies: a very recent study performed on 3T3 cells on a microfluidics platform shows how IκBs and A20 are key to make NF-κB respond to differences in the concentrations of environmental cytokines and provide a sort of activation memory that, again, was reproduced by mathematical models including both feedbacks (67).

Along these lines, a clever combination of computer vision, live cell imaging and computer modeling revealed that the levels of IκBα relative to p65 determine the responsiveness of NF-κB to TNF-α in 3T3 cells (68). We have proposed that the overall transcriptional differences in the expression of regulatory elements upon TNF-α stimulation are a major driver of the differences in the response of cells to stimuli (69), which suggests that the dynamical variation of the levels of the negative feedbacks can produce distinct NF-κB dynamics. This is in line with results obtained on an easily tunable synthetic NF-κB-like circuit reconstructed in yeast, which demonstrate how different strengths in the negative feedbacks of the circuit lead to different dynamics (70). Live cell imaging also allowed to identify novel positive feedbacks on NF-κB: when stimulating RAW cells with LPS, the total p65 fluorescence intensity increases, which reveals a positive feedback loop on p65 expression mediated by the protein Ikaros (57). Negative feedbacks do not only influence the dynamical response, but can also play a role in tuning the cell-to-cell heterogeneity: Paszek and collaborators (40) showed in SK-N-AS and MEFs – with the aid of modeling and genetic knock-outs– that the transcriptional feedback of IκBε was delayed relative to that of IκBα, and such delay maximized the oscillatory heterogeneity in the cell population. This striking result was well reproduced by a mathematical model where the feedbacks had a delay and a stochastic gene activation component. It was proposed that this maximization of diversity can lead to population robustness (40); from a purely theoretical point of view, this work provides an interesting example of how an apparent redundancy in a genetic circuit (such as the coexistence of two similar negative feedbacks) can lead to an unexpected dynamical behavior.

Not only does live cell imaging provide important clues on downstream regulators of NF-κB activity, but also of how it responds to temporally complex external stimuli. As mentioned above, the ability to manipulate the cell’s environment coupled with live cell imaging has provided unprecedented insights on how the NF-κB system translates dynamic environmental signals into dynamic intracellular signals. For example, a microfluidic device producing very short TNF-α (1-2 minutes long) pulse showed that these were already able to activate NF-κB in HeLa cells but led to a significantly smaller area under the curve compared to constant stimuli. This leads to different balances between the pro (caspase8-mediated) and anti- (NF-κB mediated) apoptotic branches downstream the TNF-α receptor and consequently to different rates of cell death (71). Interestingly, a similar device allowed also to generate ramp-like TNF-α profiles (72) and analyze the resulting NF-κB dynamic response; this device illustrated a wider variety of modes of activation and how mathematical models of the negative feedbacks’ variability could recapitulate them.

Microfluidic devices can produce periodic cytokine profiles, which have been conjectured to arise by single cytokine-emitting cells within a tissue (73) and can be informative to probe the response of NF-κB to temporally complex stimuli. In their paper Kellogg and Tay (53) showed that 3T3 cells, which behave as sustained oscillators if a flow of fresh TNF-α is continuously administered to the cells, would synchronize their oscillations 1:1 to a “sawtooth like” TNF-α periodic stimulation (obtained by periodically “refreshing” the TNF-α in the cell chamber and letting it degrade) for most of the frequencies, but not for others. This made the authors suggest that the phenomenon observed was the so-called entrainment, by which an oscillator synchronizes to an external periodic forcing if it has a period that falls in a range around its own oscillatory period, where the amplitude of the range increases with the amplitude of the external forcing. Interestingly, the range was larger than predicted by mathematical models and this was attributed to the noise inherent to NF-κB circuit, as to any genetic circuit; this idea was confirmed through mathematical modeling where intrinsic transcriptional noise was explicitly modeled in the negative feedbacks. A subsequent study (74) showed that indeed NF-κB dynamics on the same cells could switch between different “period-lockings” on time if monitored for sufficiently long time for weaker perturbation, further sustaining the notion that intrinsic noise plays a role in the way in which NF-κB dynamics responds to external time-varying stimuli. On the other hand, using commercial microfluidic systems, we showed that mouse embryonic fibroblasts that behave predominantly as damped oscillators (with a definite number of oscillatory peaks even under a flow of fresh TNF-α) would synchronize one-to-one to periodic stimulus with TNF-α for frequencies to which 3T3 fibroblasts would not, for example a period of 60 minutes (48). This again was reproduced by a relatively simple model in which NF-κB is a damped and not a sustained oscillator. Overall, these findings provided unprecedented insight on the ability of NF-κB to adapt to temporally complex external signals, and how even slightly different cells might adapt through different dynamical mechanisms to the same time-varying external signals.

Finally, live cell imaging has also contributed to enrich our knowledge on how signal transducers produce different patterns of NF-κB activation. For example, a combination of live cell imaging with selective knock-outs showed that the Trif and Myd88 branches downstream the TLR4 pathway contributed differently to the NF-κB dynamics and cell heterogeneity in RAW cells (75). Cells are indeed able to encode (at least partially) in NF-κB dynamics the presence of either LPS, TNF-α or both together (76). Other studies have shown intriguingly that cells respond to TLR2 and TLR4 activating stimuli in distinct ways but respond to their combination as if only one was present, indicating that NF-κB dynamic stimulus-specificity depends on the stimulus considered (77). Taking advantage of optogenetic tools to activate independently and selectively the MyD88 and Traf6 nodes downstream IL-1β activation and imaging NF-κB dynamics in the same cells, it was possible to identify a critical role of IRAK1 in regulating the distinct dynamics of NF-κB upon IL-1β with respect to TNF-α, which can be understood as a way for the cell to discriminate between the two signals (78). Finally, in recent work it was possible to track and quantify the dynamic formation of IKK complexes simultaneously to that of nuclear localization dynamics of NF-κB to identify what determines the response to IL1-β and TNF-α, identifying a so-called “stochastic-pooling” mechanism (79). Taken together, the above works show the growing availability of technologies and models that allow to describe how NF-κB dynamics contributes to encode simultaneously a variety of complex extracellular stimuli.

## Insights on NF-κB Control of Transcription: A Dynamic Control of Gene Expression

The central role of NF-κB in the immune response and inflammation probably relies on its transcriptional control over the production of cytokines and chemokines. In this context, live cell imaging-based studies have led to some crucial insights on the role of NF-κB dynamics in the control of gene expression. The very first observations of the oscillatory nature of NF-κB in single living cells (24) already showed that target genes could be expressed with different kinetics in populations of oscillating cells. This view was further tested by Sung and collaborators (80) who classified the gene expression dynamics of genes upon TNF-α stimulation in early (peaking at about 1h), intermediate (at about 2-3 h) and late (4 hours or later), while disruption of the NF-κB oscillatory dynamics by drugs drastically altered gene expression levels. Using similar settings two more works (42, 61) showed that the target gene expression correlated well with the fraction of activated cells in a population upon different doses of TNF-α.

Since the dynamics of NF-κB is extremely heterogeneous at single-cell level, extracting quantitative links between dynamics and transcription using population-level transcriptional assays is challenging. However, some of the methods to control NF-κB dynamics described above have contributed to provide additional insights on the control of gene expression by NF-κB. For example, in (38) it was found that pulsed TNF-α stimulation also affected gene expression levels in a target-specific way, providing an additional and intriguing link between NF-κB dynamical features and target gene expression. On the other hand, periodic sawtooth-like TNF-α profiles that can entrain NF-κB oscillations lead to higher expression levels of the target genes (53). Using microfluidics coupled with population-level microarray gene expression data, we found that periodic stimuli that synchronize NF-κB dynamics to different oscillatory behaviors lead to different dynamic patterns of gene expression at population level ranging from oscillatory to non-oscillatory mRNA expression dynamics (48). Such kinetics could be reproduced accurately in a model where the gene activation depends linearly on the concentration of NF-κB, but mRNA degradation rates were faster in early than in late genes.

More recently, the study of NF-κB dynamics upon activation with a wider variety of pathogen-associated molecules and cytokines has provided additional important clues on how NF-κB controls gene expression. In a recent work on fibroblasts and macrophage-like cell lines, it was shown that distinct TLR-activating pathogen-derived stimuli lead to distinct dynamics and activation of target gene expression, suggesting a cell specific encoding of the stimulus by NF-κB dynamics (60). Machine learning allowed to establish a connection between the different dynamics induced by a panel of pro-inflammatory stimuli in primary macrophages and the resulting target gene expression, showing that the specificity arises through the combination of different “signaling codons” (understood as fundamental features of NF-κB dynamics) emerging for each of the stimuli (59). A recent related study shows that an additional layer of complexity might further influence how NF-κB controls gene expression: using again knock-in primary macrophages (46), a mathematical model reproducing the competition between NF-κB and the histones in binding to DNA was able to reproduce NF-κB dynamics-dependent chromatin changes in enhancers that were observed experimentally, and generate long-term transcriptional memory effects.

Finally, an alternative way to gain insights on the connection between NF-κB dynamics and gene expression is through single-cell assays of the expression of target genes. The fluorescent tagging of IκBα (24) provided a first insight on how NF-κB oscillations produce oscillatory IκBα levels. An mCherry reporter under the control of the TNFA promoter gene in fluorescently NF-κB tagged RAW cells (57) allowed to show that the maximum nuclear occupancy correlates with the reporter expression. A more recent study (81) using a HIV-LTR promoter (that carries binding sites for NF-κB) controlling the expression of a destabilized GFP transgene allowed to infer that TNF-α induced transcription occurs in bursts that are ultimately driven by fold-change increase of NF-κB nuclear localization upon stimulus relative to the basal level in inactive cells, amplified by a TAT-mediated positive feedback loop. In RAW cells upon LPS stimulation it was also possible to measure NF-κB dynamics and TNF-α secretion in the same cell, showing a remarkable correlation and suggesting a role for the post-transcriptional regulator TRIF in modulating TNF-α expression (82).

Although informative, protein-based reporters have some limitations due to the lack of temporal resolution that they offer (considering the expected delay between nuclear NF-κB localization, gene transcription and translation) and, more importantly, due to the stochasticity in transcriptional activity at single gene level. Hence, measuring the expression of mRNA in target NF-κB genes can provide a more direct quantitative insight on the relation between NF-κB dynamics and transcriptional control in single cells. Using HeLa cells with a carefully controlled ratio of (fluorescently tagged) ectopically and endogenously expressed p65 and measuring its nuclear localization dynamics upon TNF-α, it was found that the heterogeneous expression of target genes assessed by single molecule RNA-FISH (smRNA-FISH) mostly depend on the fold-change in the nuclear concentration of NF-κB during the first activation (39). This finding was confirmed for a wide range of gene expression levels and for lymphocyte-derived Jurkat cells (83); such dependence has been linked through mathematical modeling to the existence of an incoherent feedforward loop (84) involving a competitive binding with a repressive transcription factor, presumably the p50:p50 homodimer. A similar smRNA-FISH approach on two target genes (NFKBIA and TNFA) in single RAW cells upon LPS, for which NF-κB was also fluorescently tagged, show that transcription levels of two genes within a single cell can be correlated (43), although the precise relation between NF-κB dynamics and transcriptional output has not been fully elucidated. Another interesting approach to probe genome-wide gene expression in target genes is single-cell RNA-seq which, combined with live cell imaging of the same single RAW cells (58) showed that different types of NF-κB dynamics lead to distinct patterns of gene expression that could be linked with the distinct dynamic interaction between NF-κB, gene promoters and enhancers, although such interaction was not explicitly modeled. More recently, the transcriptional bursts induced by NF-κB activation have been characterized. The direct observation of the dynamics of nascent transcription using the MS2 system alongside that of NF-κB localization in single living HeLa cells showed that nascent transcription is surprisingly prompt and sharp as compared to NF-κB activation for a fraction of first responders (55). Interestingly, a mathematical model that combined NF-κB-driven activation of the gene through a multi-step activation process with a refractory time was able to reproduce for a wide range of parameters the prompt and sharp nascent transcriptional response, further hinting to the potential emergence of novel dynamics as a result of the combination of NF-κB and certain gene-activity motifs. However, a correlation between NF-κB dynamical features and transcription bursts at single cell level was not found. Of note, in a wider panel of target genes a correlation between the mathematically inferred burst size and TNF-α dose was found, which presumably might hold true also for levels of NF-κB activation, although this has not been assessed (85).

Taken together, the above results point to an important role of NF-κB activation dynamics in determining target gene expression. However, we lack universal models connecting NF-κB dynamics and transcriptional output, but this can be due to a number of factors that for sure play a key role alongside NF-κB dynamics in target gene expression: the use of different cell types and target genes with different degrees of chromatin accessibility, different NF-κB tagging strategies, the role of other pathways activated in parallel by the same stimuli and the complex interplay between TF dynamics and epigenomic regulation. We discuss these issues in further detail below.

## Conclusions and Future Perspectives

### An Emerging Dynamic View of the NF-κB System

**Figure 3:**
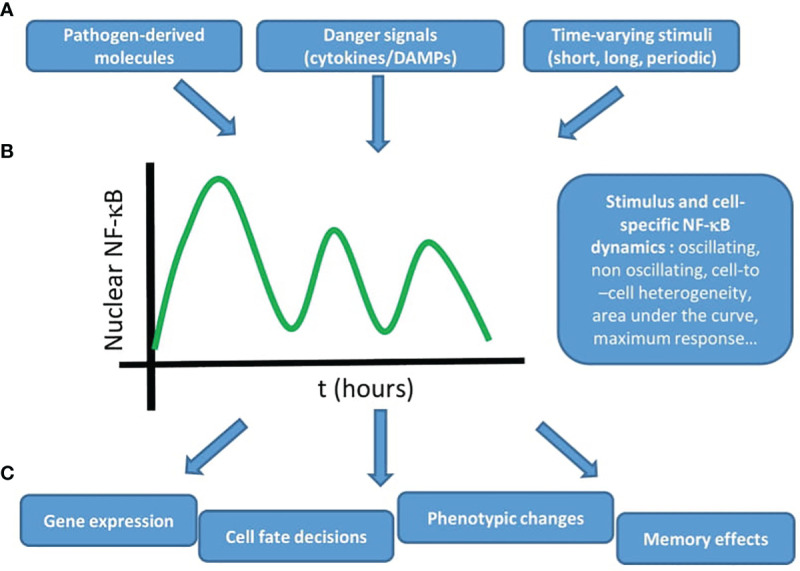
The NF-κB system uses dynamics to produce adequate outputs upon different stimuli. The emerging view from live cell imaging studies on the dynamics of NF-κB is that **(A)** different stimuli, derived from pathogens or secreted by cells, potentially in a time-varying fashion **(B)** lead to stimulus specific NF-κB dynamics (with a certain degree of cell-to-cell heterogeneity) that **(C)** produce different outputs, such as gene expression, cell fate decisions, phenotypic changes, and memory effects. Taken together, these studies underline the importance of NF-κB dynamics to understand its role in the context of inflammation and the immune response.

In this review we have tried to provide a panoramic view on the growing research field that attempts to provide a deeper characterization of the NF-κB system using live cell imaging. The emerging view of these studies is summarized in **Figure 3**: these studies show how different stimuli (**Figure 3A**) like cytokines and pathogen-associated molecules lead to distinct NF-κB dynamics in a stimulus- and cell-specific manner (**Figure 3B**). As a result, different cellular outputs are obtained (**Figure 3C**) ranging from different patterns of gene expression (with characteristic kinetics or abundances), different cell fate decisions (e.g. proliferation versus apoptosis), changes in the epigenome and even memory effects (e.g. cells responding differently to consecutive stimuli). Of course, we are far from a complete picture of how this dynamic signal processing takes place, but we think that the works presented here make a solid case for the importance of adopting a dynamic point of view when trying to understand how NF-κB is involved in different biological processes.

This dynamical point of view can be particularly fruitful in immunological studies. For example, a recent high-throughput unbiased screening shows the key role that is played by NF-κB in primary human T-cell responses (13), so key elements of the NF-κB regulatory circuit are found to be fundamental for cytokine production. A next reasonable step would be to study what is the activation status of NF-κB upon selected T cell- activating stimuli; indeed, the study of nuclear localization of NF-κB is now amenable using flow cytometry approaches (86). However, in the light of what we discussed here, this might yet prove insufficient since it would be a simple “on-off” characterization of NF-κB (when we have seen that different stimuli will not just “activate” the system, but make it evolve temporally in different ways). Hence, these studies could be complemented with a characterization of the dynamics of NF-κB in the cells for certain conditions of interest; indeed, a similar dynamics-based workflow was recently used on cell lines and allowed to identify novel NF-κB regulators (87) and to identify drugs targeting the NF-κB response (88), and could for sure complement other unbiased approaches in gaining deeper insights on the role played by NF-κB in immune cells.

### From Adding Complexity to the NF-κB System to Understanding Its Complex Behavior?

We believe that the examples discussed in this review show that we do not only need to identify new players that add complexity to the NF-κB regulatory system to understand how it works. Instead, we might also need to better understand the complex behaviors that this complex genetic circuit can display in different situations. Of note, most of the key players of the NF-κB system were identified years ago (3); for sure, we cannot exclude that new elements of the regulatory circuit will be identified, but this is unlikely to change the general picture. The works here presented provide a clear example of how a deeper characterization of the system (in this particular case, understanding the role played by NF-κB dynamics in different situations) can contribute to explain how this system carries out the wide variety of functions that are attributed to it. We believe that this shift in the point of view (from adding complexity to our picture of a genetic circuit to having a deeper understanding of the complex behaviors that it can display) is already taking place and will probably become dominant once the structure of most genetic circuits will be completely determined, and the (nontrivial) remaining task will be to understand how they work.

### The Need of Standardization, From Live Cell Imaging Data to Modeling

The above discussion can also be framed within a wider trend towards the use of more quantitative approaches to characterize how genetic circuits work. To achieve this goal, there is a pressing need to standardize approaches and methodologies. In case of the NF-κB system, availability of imaging data is limited and mostly relies on the willingness of individual researchers to share their data. This is probably related with the fact that imaging datasets are typically heavy and costly to maintain in online repositories, so coordinated institutional efforts in this direction would be necessary, as for genomics data. A more efficient data sharing would allow for an uniformization of data acquisition and analysis by individual research groups, making results easier to compare between laboratories. This could also lead to a major leap forward in the mathematical modeling efforts: currently there is a wide variety of mathematical models that are able to qualitatively reproduce the dynamics of the NF-κB system in conditions of interest, but seldom two publications (unless they come from the same lab) share the same mathematical model, among other reasons because typically each model is tailored to address specific questions in very well defined experimental conditions. Making available a wider variety of data would allow the implementation of unified mathematical models whose validity could be measured by how well they can reproduce a wide variety of live cell imaging data from different groups. These models would also allow us to account for cell and context specific variability in a quantitative way (e.g. through differences in different parameters/parameter combinations) that can then be further tested and verified through experiments.

### Towards a More Quantitative Biophysical Model of NF-κB Regulation

The efforts above must be also accompanied by a more detailed biophysical characterization of NF-κB regulation. As pointed out above, most of the mathematical models share a common regulatory core and rely on biochemical rates that only seldom were estimated in the same conditions for the same cells. We believe that the increasing ability in producing knock-ins, knock-outs and synthetic versions of the NF-κB system (70) combined with live cell imaging approaches might soon make it possible to provide cell-specific measurement of key parameters of the system. However, we cannot exclude that novel mechanisms should be taken into account. For example, we have a limited knowledge on the actual fraction of NF-κB molecules that are bound to relevant regions of the genome in each amount of time (89), which might influence their nuclear-cytosolic relocation. Another important mechanism that has only been characterized for the inhibitors is the molecular stripping by which IκB molecules bind and actively detach DNA-bound molecules (90). We cannot exclude other interactions influencing NF-κB dynamics, as recently revealed by high throughput screenings showing that mediator complex subunits *MED12* and *MED24* have a negative impact in NF-κB nuclear localization (87). Finally, it is also plausible that mechanical cues and in particular changes in the cell’s cytoskeleton might influence the overall NF-κB cytoplasm-to-nucleus transport process, as demonstrated for actomyosin (91).

### A Universal Model of NF-κB Driven Transcription?

In spite of the wide variety of studies that we have described that correlate NF-κB dynamics and transcription, we also lack a universal quantitative model to link NF-κB nuclear localization dynamics and transcriptional dynamics. By far, the models correlating fold-change of nuclear NF-κB with mRNA expression levels are the ones leading to the most accurate predictions at single cell level (39, 81, 83), but only few studies have been able to confirm this model or to provide alternative models with comparable power. This might be because many correlative studies use ectopic expression of fluorescently tagged NF-κB, where the ratio with the endogenous might vary from cell to cell, which makes it difficult to establish these quantitative links. But there might be deeper biological reasons for this. As sketched above, NF-κB dynamics are often cell-specific and the same might apply to its control of gene expression, potentially in a gene-specific way. Related to this, it is widely accepted that NF-κB mediated gene activation heavily relies also on the epigenetic context (4) and this picture is further complicated by the fact that NF-κB dynamics itself might affect the epigenome (46). In this line, a recent study from our lab shows that relatively uniform nuclear localization NF-κB dynamics across a population can produce quite different bursting transcriptional dynamics in a nascent transcription reporter (55); this suggests that other factors might influence gene activation, but it might also well reflect the complex interaction at a molecular level between NF-κB and the gene promoter. However, we know very little about how NF-κB behaves at single molecule level, where interactions with DNA seem very short-lived (89) and are shaped by different dimerization behaviors within the nucleus (92). Hence, it might be just not possible to provide a fully quantitative model connecting NF-κB nuclear localization dynamics with gene expression, since it might be unable to capture the complexity of NF-κB multi-scale dynamic interaction with the genome. This can only be clarified by further studies in which transcription and NF-κB dynamics can be measured precisely in single cells under very controlled settings.

### Towards a Characterization of NF-κB Dynamics in Primary Cells… and Tissues?

Many NF-κB studies have been performed in immortalized cell lines that might carry transformations that lead to distortions in NF-κB signaling. As discussed above, this might be aggravated by the use of ectopically expressed fluorescently tagged NF-κB, whose dynamics might not reflect the endogenous one, although of course this is something that can be controlled for. However these difficulties might be circumvented by the increasing availability of mouse models in which the endogenous p65 has been fused to fluorescent molecule such as GFP (44), Venus (59) and mScarlet (92) among others. This would first allow to characterize the role of NF-κB in primary cells, which is strongly cell-type specific even for cells within the same tissue (93). But a second related and indirect advantage is that this might allow to study NF-κB behavior in cells within the truly physiological context: the tissue. Most of the studies cited hereby deal with cells that have been cultured in 2D and that are subjected to a strong dose of inflammatory stimulus. Instead, cells within a tissue interact with a complex environment and potentially much more complex mixture of signals at concentrations potentially very different to those used *in vitro*. Indeed, some progress is being made in trying to characterize the dynamics of NF-κB in cell-to-cell communication in standard 2D cultures (41) or in microfluidic devices (94, 95), or in mathematical models of inflammatory signal propagation in tissues informed by single-cell live cell imaging data (43). However, we believe that imaging living NF-κB dynamics in cells within their tissue context will become possible in the next few years, as already exemplified (96), and will allow us to gain additional understanding on the role of NF-κB and its dynamical function in physiological and pathological situations.

### A Dynamical View of the Whole NF-κB System

Most of the works discussed here focus on the activation of the canonical NF-κB pathway upon stimulus, involving p65. However, much less is known about the dynamics of other members of the NF-κB family, although some efforts have already been performed in this direction. A recent study performed on cell lines expressing fluorescently tagged p65 and c-Rel show that each subunit displays distinct dynamics (with p65 more prone to oscillate) that are key to determine target gene expression (60). It is also important to notice that NF-κB is dimeric and the same group reported that p65:p65 dimers are more abundant than what might be expected (92). Finally, the noncanonical branch of NF-κB remains a huge area of research for exploration using the methodologies that we have described here. We expect that they might lead to important insights in the field of cancer research, since a wide variety of NF-κB-related cancers display mutations in the regulatory circuit of both NF-κB branches (97).

### Crosstalk Between NF-κB and Other Pathways, in Health and Disease

As pointed out, NF-κB is key in the immune response but it is also well known that most of the stimuli that activate NF-κB would lead to an activation of other inflammatory pathways. Indeed, some efforts have also been applied to understand how the dynamics of NF-κB, MAPK and interferon signaling interact to provide an adequate cell response upon TLR-activating signals (75, 78, 98); however, a more detailed imaging-based characterization of this “inflammatory crosstalk” is fundamental and should also reach other important players in inflammation, such as members of the STAT family (99). But we also have to consider situations in which different and not necessarily “inflammatory” pathways are activated simultaneously to NF-κB by different stimuli. An example is NF-κB and p53, two signaling pathways that are typically activated in cancer and for which a crosstalk has been defined using biochemical “static” approaches (100). Indeed there is already live cell imaging evidence showing that NF-κB activation might disrupt p53 signaling upon gamma irradiation (101), and this could presumably alter the cancer cell’s life-death decision upon treatment, which has been shown to rely on p53 dynamics (29). A dynamical point of view to characterize the crosstalk with crucial cancer-related pathways could indeed be fundamental to enrich our view of the role of NF-κB in cancer biology (97), but also in the complex interplay between cancer and inflammation (21) and in the wide variety of pathologies that have been shown to involve NF-κB (6), including auto-immune diseases (102).

In sum, the live cell imaging centered approach to study NF-κB has shown the importance of dynamics in understanding the function of this fundamental genetic circuit in the immune response. We believe that this kind of approach will continue to reveal itself fundamental to dissect the role of NF-κB within different cell types, in their tissue context, and its interaction with other pathways both in physiological and pathological processes.

## Author Contributions

Conceptualization: SZ. Supervision: SZ, MB. Writing-Original draft: SZ, CK. Writing-review and Editing: SZ, CK, MB. Funding acquisition: SZ, MB. All authors contributed to the article and approved the submitted version.

## Funding

The research leading to these results has received funding from AIRC under IG 2020 - ID. 24702 project – P.I. MEB, from Ospedale San Raffaele (OSR Seed Grant to SZ) and Vita-Salute San Raffaele University (predoctoral fellowship to CK).

## Conflict of Interest

The authors declare that the research was conducted in the absence of any commercial or financial relationships that could be construed as a potential conflict of interest.

## Publisher’s Note

All claims expressed in this article are solely those of the authors and do not necessarily represent those of their affiliated organizations, or those of the publisher, the editors and the reviewers. Any product that may be evaluated in this article, or claim that may be made by its manufacturer, is not guaranteed or endorsed by the publisher.
